# Practice among Healthcare Workers Regarding the Use of Respiratory Protective Equipment during COVID-19 Pandemic in a Tertiary Care Center: A Descriptive Cross-sectional Study

**DOI:** 10.31729/jnma.5500

**Published:** 2021-04-30

**Authors:** Sani Sipai, Bishnu Rath Giri, Sudhir Sapkota, Ram Hari Chapagain, Santosh Manandhar, Sudip Chandra Subedi, Binod Dahal

**Affiliations:** 1 Department of Pediatrics, National Academy of Medical Sciences, Kanti Children's Hospital, Maharajgunj, Kathmandu, Nepal

**Keywords:** *face masks*, *healthcare workers*, *practice*, *respiratory protective equipment*

## Abstract

**Introduction:**

Healthcare workers are always at the risk of exposure to different diseases like respiratory illness including COVID-19. Using appropriate face mask or respiratory protective equipment correctly can prevent transmission of diseases from and to healthcare workers while caring for patients. The study aimed to find out the practice regarding use of face masks during the COVID-19 pandemic in a tertiary care center.

**Methods:**

A descriptive cross-sectional study was conducted at a tertiary care hospital during June-July 2020 after receiving ethical approval from the review committee regarding practice of use of face masks. Convenience sampling method was used and a sample size of 162 was taken. Descriptive statistical analysis was done. Point estimate at 95% Confidence Interval was calculated along with frequency and proportion for binary data.

**Results:**

Among 162 participants, 123 (75.9%) knew the correct way of using the masks (72.5-79.3 at 95% Confidence Interval).

**Conclusions:**

In this study regarding practice of use of face masks, most of the healthcare workers knew the correct way of using masks and practised hygiene before and after using masks.

## INTRODUCTION

Healthcare workers (HCWs) are at high risk of various respiratory hazards including coronavirus disease-19 (COVID-19). World Health Organization (WHO) recommends use of medical mask for all HCWs working in an area with community transmission of COVID-19 and respirators for those working in aerosolgenerating procedures.^1^ Center for Disease Control (CDC) recommends N95 (or equivalent respirators) or surgical masks to prevent infection to and from HCWs while taking care of patients with suspected or proven disease. All HCWs need to be trained for appropriate use of Respiratory Protection Equipment (RPE).^2,3^

There are few studies about the practice of HCWs regarding the use of RPE especially from developing countries. These studies highlight the deficient supply and improper use of RPE.^4-6^ The search of literatures doesn't reveal any such study from Nepal.

Hence, the study aimed to know the practice regarding use of face masks during the COVID-19 pandemic in a tertiary care hospital of Nepal.

## METHODS

This was a descriptive cross-sectional study conducted among the healthcare workers including doctors, nurses, paramedical staff and laboratory and medical technicians at Kanti Children's Hospital (KCH), a tertiary care children's hospital in Kathmandu, in June-July 2020. During the study period, due to the increasing number of COVID-19 cases in the country, there was a lockdown imposed by the government and HCWs were advised to manage duty alternately to minimize exposure in the latter half of the study period when the lockdown was partially relieved. However, the daily new diagnosis of COVID-19 was on the rise and there was no rampant community transmission. There were few kinds of literature and diagnostic facilities available. Ethical approval for this study was obtained from the institutional review committee at KCH (Ref. no. 842) and informed written consent was taken. All healthcare workers (HCWs) working at the study site, who consented to participate, were included in the study. Healthcare workers working part-time or visiting from other institutes for practices or observership were excluded A convenient sampling method was used and a sample size of 109 was calculated, considering the margin of error at 6% and confidence interval of 95%, and 88.5% as the correct knowledge and practice of using face masks in the previous study.^6^

The sample size was calculated using the formula:

$$\begin{array}{ll} \text{n} & ={\text{Z}}^{\text{2}}\times \text{p}\times \text{q}/{\text{e}}^{\text{2}}\\ & ={\left(1.96\right)}^{2}\times 0.885\times 0.115/{\left(0.06\right)}^{\text{2}}\\ & =109 \end{array}$$

where,

n = sample size
Z = 1.96 at 95% Confidence Interval (CI)
p = prevalence of previous study, 88.5%^6^
q = 1-p = 1-0.885 = 0.115
e = margin of error, 6%

Taking 10% non-response rate, the total sample size becomes 120. However, the total sample taken was 162. The study was conducted by interview using a structured questionnaire. The questionnaire was developed after a review of previous literature on the proper use of surgical face masks and the guidelines of the CDC.^2,6,7^ The questionnaire included basic demographic characteristics (age, gender, job designation), and knowledge, and practice related questions regarding the use of a face mask to limit COVID-19 exposure. During sample collection, some participants did not respond to some of the questions leading to missing data so a valid percentage was considered while finding out results. The information obtained from the participants was entered and calculated using Statistical Package for the Social Sciences (SPSS) Statistics for Windows, Version 20.0. Mean with standard deviations were calculated for age and frequency with percentages for responses with categorical variables.

## RESULTS

A total of 162 participants were included in the study. One hundred twenty-three (75.9%) (72.5-79.3 at 95% Confidence Interval) knew the correct way of wearing a surgical mask. On the question about areas required to be covered by surgical masks, 151 (93.2%) answered correctly (nose, mouth and chin). One hundred fifty eight (97.5%) HCWs had the practice of putting on masks while going out and had a practice of wearing masks on hospital premises as well. About 156 (96.3%) practised hand hygiene before and after using face masks. One hundred twenty-five (77.2%) of participants reused the face masks.

The mean age of participants was 32.78±8.55 years. Among the participants 66 (40.7%) were doctors, 72 (44.4%) were nurses, 7 (4.3%) were laboratory staff, 2 (1.2%) were physiotherapists and 6 (3.7%) were others. The median work experience of the participants was 60.0 months. Among them 106 (65.4%) participants were directly involved in the care of suspected or proven COVID-19 cases.

Fifty-two (32.1%) responded eight hours as the maximum duration for wearing a face mask with no compromise in safety. About 147 (90.7%) of participants believed face masks can prevent transmission of respiratory disease like COVID-19. About 58 (35.8%) of participants believed that both surgical and N95 masks or equivalents are equally effective in preventing respiratory illness like COVID-19.

Out of 162 respondents, 125 (77.2%) of participants used one mask for the whole day, either new or reused one. About 126 (77.8%) used surgical masks while 36 (22.2%) used N95 or equivalent masks but no participants used fabric masks. Five (3.1%) of them used to remove the mask but 155 (95.7%) didn't while talking to patients and visitors. About 84 (51.9%) touched face masks/RPE 1-5 times while 35 (21.6%) touched 5-10 times and 29 (17.9%) touched more than 10 times in a shift but 13 (8.0%) didn't touch their RPE in a shift. Seventy-four (45.7%) took off face masks/ RPE 1-2 times while 55 (34.0%) took off 3-5 times and 14 (8.6%) took off more than 5 times in a shift but only 12 (7.4%) didn't take off their RPE in a shift.

Meanwhile, 117 (72.2%) of participants stored RPE before reuse. About 45 (27.8%) stored N95 masks, 27 (16.7%) stored surgical masks while 75 (46.3%) stored both masks but 13 (8.0%) didn't store. Fifty-four (33.3%) stored surgical masks and 67 (41.4%) stored N95 masks for 2-3 days before reuse.

Ninety-four (58.0%) reused surgical masks consecutively for 1-3 days, 20 (12.3%) for 3-7 days and 46 (28.4%) did not use a single mask consecutively for more than one day. While 51 (31.5%) reuse N95 masks consecutively for 1-3 days, 70 (43.2%) for 3-7 days and 10 (6.2%) did not use the mask consecutively for more than one day.

For disinfection of mask, 58 (35.8%) of them used sunlight exposure, 38 (23.5%) stored, 26 (16.0%) washed face masks but 18 (11.1%) did not practise disinfection. About 150 (92.6%) of participants removed face masks from behind the ear or head. During disposal of used face masks, 92 (56.8%) used a closed lid bin while 67 (41.4%) used an open dustbin and 2 (1.2%) disposed of open (Table 1).

**Table 1:** Practice of re-using and disposal of face masks among HCWs.

Statement	Response	n (%)
Do you practice Reusing face masks?	Yes	125 (77.2)
Average no. of days a surgical mask reused consecutively	1 day	46 (28.4)
	1-3 days	94 (58.0)
	3-7 days	20 (12.3)
Average no. of days a N95 mask reused consecutively	1 day	10 (6.2)
	1-3 days	51 (31.5)
	3-7 days	70 (43.2)
	>7 days	27 (16.7)
Do you practice storing face masks	Yes	117 (72.2)
	No	41 (25.3)
Which type of face masks do you store?	N95 or equivalents	45 (27.8)
	Surgical mask	27 (16.7)
	Both	75 (46.3)
	I don't store	13 (8.0)
Average no. of days a surgical mask is stored before reuse	1 day	52 (32.1)
	2-3 days	54 (33.3)
	3-5 days	29 (17.9)
	>5 days	22 (13.6)
Average no. of days a N95 mask is stored before reuse	1 day	24 (14.8)
	2-3 days	67 (41.4)
	3-5 days	38 (23.5)
	>5 days	28 (17.3)
Response regarding reusing face masks	Inadequate supply	141 (87.0)
	It is safe to reuse	9 (5.6)
	Disinfection is quite effective	4(2.5)
	Others	6 (3.7)
What is your practice of RPE disinfection?	None	18 (11.1)
	Storage	38 (23.5)
	Sunlight	58 (35.8)
	Washing	26 (16.0)
	Spraying spirit	1 (0.6)
	Others	19 (11.7)
How do you dispose of used RPE?	In a dustbin	67 (41.4)
	In a closed lid bin	92 (56.8)
	Open disposal	2 (1.2)
	I don't know	1 (0.6)

## DISCUSSION

From developing countries, very few studies on the subject have been published.^3,6^ This study aimed to know the practice of HCWs on the use of RPE. The results indicated that most of the studied HCWs had good practice regarding the use of RPE. All the participants who responded had the practice of putting on masks while going out and in hospital premises. This study showed 75.9% of the participants knew the correct way of wearing a surgical mask and 93.2% knew about areas required to be covered by surgical masks. As compared to the study in Pakistan by Kumar J, and et al.^6^ showed, 88.5% of participants knew the proper steps of wearing a surgical face mask. The correct way of wearing a surgical mask is by facing out the coloured side independent of health status.^8^

In the study by Wada K and et al.^9^ participants wearing a face mask were reported to be practising additional preventive hygiene measures while in this study, 96.3% practised hand hygiene before and after using face masks.

This study found that only 3.1% of them used to remove the mask while talking to patients and visitors, but 51.9% touched face masks/RPE 1-5 times in a shift and 45.7% took off face masks/RPE 1-2 times in a shift while in the study by Kumar J et al.^6^ found 13.8% of them removed the mask to talk with patients.

The proportion of reuse is very high. We found 77.2% of participants reused the RPE (face masks) and 72.2% stored RPE before reuse; 27.8% stored N95 masks, 16.7% stored surgical masks and 46.3% stored both masks.^10^ As compared to study by Kumar J et al.^6^ found only 20.2% reused face masks. Although statistically not significant, 69.7% of doctors as compared to 83.2% of other staff reused face masks. The reason for more than 85% reuse is decreased supply.^11^

Consecutive use is not safe but 58% of our HCWs are practicing consecutive use. People tend to use N95 or equivalents for more days consecutively than surgical masks.

A colour-coded bin system is recommended for the proper disposal of biomedical waste in hospitals.^12^ Kumar J et al.^6^ found 44.9% disposed of in the yellow-coded bag for disposal of face mask; while our study found that 56.8% used closed lid bins for disposal.

The limitations of this study include the cross-sectional nature of the study design limited to one hospital with a small sample size. Further studies should be carried out on a larger sample size so that the results could be generalized.

## CONCLUSIONS

The practice of the use of respiratory protective equipment or face masks among the healthcare workers at a tertiary care children's hospital was found that HCWs knew the correct way of using masks and areas to be covered by masks. HCWs practised hygiene before and after using face masks. However, due to inadequate supply, they were reusing the face masks. All the participants who responded had the practice of putting on masks while going out and when on hospital premises. It was found that very few participants used to remove the mask while talking to patients and visitors, but around half of them touched face masks/RPE 1-5 times and took off face masks/RPE 1-2 times in a shift. More than half of the participants used a closed lid bin during the disposal of used face masks. More studies of similar nature are urgently required from different countries.
